# Spatiotemporal Patterns and Equity Analysis of Premature Mortality Due to Ischemic Heart Disease Attributable to PM_2.5_ Exposure in China: 2007–2022

**DOI:** 10.3390/toxics12090641

**Published:** 2024-08-31

**Authors:** Yanling Zhong, Yong Guo, Dingming Liu, Qiutong Zhang, Lizheng Wang

**Affiliations:** 1 School of Geological Engineering and Geomatics, Chang’an University, Xi’an 710054, China; 2 Department of Criminal Technology, Sichuan Police College, Luzhou 646000, China; 3 China Coal Aerial Photogrammetry and Remote Sensing Group Co., Ltd., CNACG (ARSC), Xi’an 710199, China

**Keywords:** China, PM2.5 exposure, ischemic heart disease, premature mortality, environmental equity

## Abstract

Long-term exposure to PM_2.5_ pollution increases the risk of cardiovascular diseases, particularly ischemic heart disease (IHD). Current assessments of the health effects related to PM_2.5_ exposure are limited by sparse ground monitoring stations and applicable disease research cohorts, making accurate health effect evaluations challenging. Using satellite-observed aerosol optical depth (AOD) data and the XGBoost-PM25 model, we obtained 1 km scale PM_2.5_ exposure levels across China. We quantified the premature mortality caused by PM_2.5_-exposure-induced IHD using the Global Exposure Mortality Model (GEMM) and baseline mortality data. Furthermore, we employed the Gini coefficient, a measure from economics to quantify inequality, to evaluate the distribution differences in health impacts due to PM_2.5_ exposure under varying socioeconomic conditions. The results indicate that PM_2.5_ concentrations in China are higher in the central and eastern regions. From 2007 to 2022, the national overall level showed a decreasing trend, dropping from 47.41 μg/m^3^ to 25.16 μg/m^3^. The number of premature deaths attributable to PM_2.5_ exposure increased from 819 thousand in 2007 to 870 thousand in 2022, with fluctuations in certain regions. This increase is linked to population growth and aging because PM_2.5_ levels have decreased. The results also indicate disparities in premature mortality from IHD among different economic groups in China from 2007 to 2022, with middle-income groups having a higher cumulative proportion of IHD-related premature deaths compared with high- and low-income groups. Despite narrowing GDP gaps across regions from 2007 to 2022, IHD consistently “favored” the middle-income groups. The highest Gini coefficient was observed in the Northwest (0.035), and the lowest was in the South (0.019). Targeted policy interventions are essential to establish a more equitable atmospheric environment.

## 1. Introduction

PM_2.5_ is a crucial component of air pollution that can enter the bloodstream through the lungs [1,2], impacting cardiovascular health. Long-term exposure to PM_2.5_ increases the risk of developing and dying from ischemic heart diseases (IHDs) [3,4], a category of conditions characterized by reduced blood flow to the heart muscle, such as atherosclerosis, coronary artery blockage, myocardial infarction, and other related conditions [5,6]. The mechanisms underlying the PM_2.5_–IHD association involve systemic inflammation, oxidative stress, endothelial dysfunction, and the promotion of atherosclerosis [7]. The inhalation of PM_2.5_ triggers inflammatory responses in the respiratory system, leading to systemic inflammation and an imbalance in the autonomic nervous system, subsequently affecting the cardiovascular system [8]. Understanding the long-term, large-scale mortality rates related to PM_2.5_ exposure is vital for formulating national air quality control and public health policies and contributes to efforts to mitigate the health impacts of environmental hazards.

Current evaluations of health effects related to PM_2.5_ exposure mainly rely on data from ground monitoring stations. However, data from fixed monitoring stations often fail to represent comprehensive spatiotemporal exposure, which can affect the accuracy of health impact assessments [9,10,11,12]. Satellite-based PM_2.5_ concentration estimates offer high resolution and broad coverage, representing PM_2.5_ exposure levels and spatiotemporal distribution more accurately than numerical models alone. Van et al. [13] pointed out that remote sensing PM_2.5_ estimates better reflect regional PM_2.5_ pollution levels than ground-based measurements. Shen et al. [14] also argued that satellite remote-sensing-based PM_2.5_ monitoring is more advantageous than ground station monitoring in studies of rural PM_2.5_ emission sources. Zheng et al. [15] analyzed the contribution of different emission sources to PM_2.5_-attributable premature deaths, finding that 32% were attributable to agricultural sources. Yu et al. [16] argued that using satellite remote sensing data combined with ground monitoring data allows researchers to more accurately assess the spatiotemporal distribution of PM_2.5_ and its health impacts, providing a scientific basis for policy-making. Yue et al. [17] analyzed the Air Pollution Prevention and Control Action Plan in China using global PM_2.5_ data from remote sensing, finding that by 2017, the policy had significantly reduced the incidence of diseases related to PM_2.5_ exposure. Liu et al. [18] showed that in regions with high PM_2.5_ concentrations such as Beijing–Tianjin–Hebei, the Yangtze River Delta, and the Pearl River Delta in China, PM_2.5_ exposure levels were positively correlated with cardiovascular disease mortality rates. However, due to disciplinary limitations, the application of remote sensing products in public health research related to long-term PM_2.5_ exposure remains limited, and quantitative studies on the health impacts of PM_2.5_ exposure based on remote sensing data are still lacking.

The risk of IHD attributable to PM_2.5_ exposure is typically quantified based on epidemiological cohort studies, which are well developed in Europe and the United States [19,20,21]. In recent years, China has initiated cohort studies on PM_2.5_-related cardiovascular diseases, but prospective cohort studies remain scarce [22,23,24], leading to challenges in accurately describing the impact of PM_2.5_ pollution on cardiovascular diseases. The Global Exposure Mortality Model (GEMM), proposed by Burnett et al. [25] in 2018, quantifies the impact of long-term PM_2.5_ exposure on mortality rates globally and in specific regions, incorporating data from various regions, including a Chinese cohort, making it suitable for evaluating the impact of PM_2.5_ exposure on IHD in China. Additionally, the growing Chinese economy and the resulting air pollution have varying impacts on different economic groups. Research by Huang et al. [26] and Zhao et al. [27] indicates that diseases and economic burdens associated with PM_2.5_ disproportionately affect vulnerable populations. According to a WHO report [28], 89% of premature deaths due to air pollution in 2019 occurred in low- and middle-income countries and regions. Premature death refers to dying before the average expected lifespan. Although many studies have investigated the health effects of PM_2.5_ in China [29,30,31], few have examined how these effects are distributed across income groups. Therefore, tracking the distribution of IHD mortality related to PM_2.5_ across different income populations is crucial for developing and monitoring targeted policies to mitigate environmental inequalities.

On this basis, the study utilizes the GEMM model based on the Chinese cohort and satellite-derived PM_2.5_ concentration retrieval results to assess premature mortality due to IHD caused by PM_2.5_ exposure in China from 2007 to 2022. Compared to station-based assessments, our method continuously depicts the spatial relationship between PM_2.5_ exposure and IHD mortality rates. Additionally, we evaluate the distribution differences in IHD due to PM_2.5_ exposure under varying socioeconomic conditions, explore the resulting environmental health equity issues, and provide scientific references for effective and targeted atmospheric governance policies.

## 2. Materials

### 2.1. Study Area

**Figure 1:**
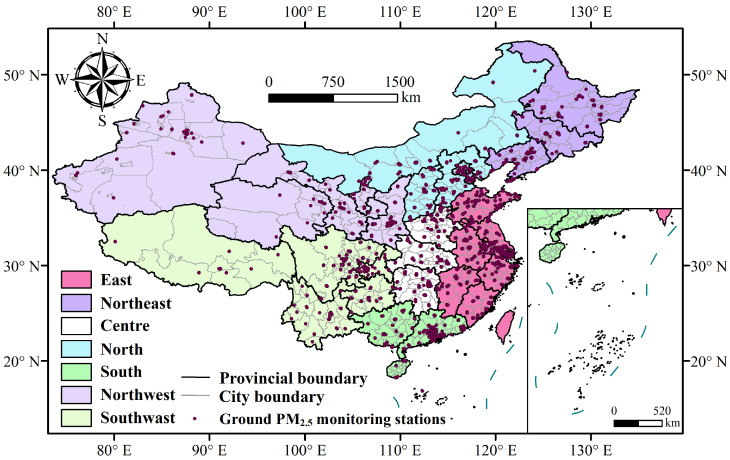
Regional division in this study.

The study area covers China, divided into seven geographic regions—Central China, North China, East China, South China, Northwest China, Southwest China, and Northeast China—considering differences in climate, topography, historical culture, and economic development (Figure 1).

### 2.2. Ground-Level PM_2.5_

Ground PM_2.5_ concentration data were sourced from the National Environmental Air Quality Monitoring Network of the Ministry of Ecology and Environment of the People’s Republic of China, accessible from the China National Environmental Monitoring Center website (CNEMC) (https://www.cnemc.cn/, accessed on 1 June 2024). The number of monitoring stations has increased from 1436 in 2012 to 2026 in 2022, as shown in Figure 1. The monitoring stations use Filter Dynamic Measurement Systems (FDMSs) and Tapered Element Oscillating Microbalances (TEOMs) to measure PM_2.5_ concentrations, calibrated and processed according to national air quality standards (GB3095-2012) [32] and environmental protection standards (HJ618-2011) [33] for quality control.

### 2.3. Satellite Data

The three types of aerosol optical depth (AOD) products from the MODerate-resolution Imaging Spectroradiometer (MODIS) aboard the Terra and Aqua satellites—Deep Blue (DB), Dark Target (DT), and Multi-Angle Implementation of Atmospheric Correction (MAIAC)—were calibrated and gap-filled. The resulting spatially continuous AOD data serve as the foundational data for PM_2.5_ retrieval. MODIS AOD products are highly accurate, with an expected error (EE) of [±(0.05 + 15%)] when validated against the Aerosol Robotic Network (AERONET) [34,35].

### 2.4. Auxiliary Data

Auxiliary data were used in conjunction with AOD data to estimate PM_2.5_ concentrations. The meteorological data, which include atmospheric boundary layer height (m), relative humidity (%), temperature (K), wind speed (m/s), precipitation (kg/m²), visibility (m), and surface pressure (Pa), were sourced from the National Centers for Environmental Prediction (NCEP) dataset. Terrain data were obtained from the Global Digital Elevation Model Version 2 (GDEM V2), which is jointly published by the Ministry of Economy, Trade and Industry (METI) of Japan and the National Aeronautics and Space Administration (NASA) of the United States. The Fine Mode Fraction (FMF) is obtained using the LookUp Table-based Spectral Decomposition Algorithm (LUT-SDA) [36,37,38] combined with Ångström derivative calculations. The datasets were standardized according to coordinate frameworks, spatial extent, resolution, and data format to ensure that all data were on the same coordinate grid with a 1 km resolution and were then used for modeling.

### 2.5. Socioeconomic Data

Gridded population data were obtained from LandScan (https://landscan.ornl.gov/, accessed on 1 June 2024), released by the Oak Ridge National Laboratory (ORNL) of the U.S. Department of Energy. LandScan combines geospatial science, remote sensing, and machine learning algorithms to provide 1 km scale global population distribution data, applied in multiple fields [39,40,41]. Population age distribution data were sourced from the National Data Center of the National Bureau of Statistics (https://data.stats.gov.cn/, accessed on 1 June 2024). GDP data were obtained from the Resource and Environment Science and Data Center of the Chinese Academy of Sciences (https://www.resdc.cn/, accessed on 1 June 2024). The GDP data were generated based on statistical GDP data at the county level, incorporating the spatial interaction patterns between land use types closely related to human activities, nighttime light intensity, and settlement density data. This was achieved by spatial interpolation of the data, which has a spatial resolution of 1 km. For years with missing data in this dataset, GDP values were estimated using Equation (1):

(1) $$GDP_{x}=GDP_{x-1}\times P_{i}$$

where $GDP_{x}$ represents the GDP of the grid in year *x*, and $P_{i}$ is the GDP growth rate of the district-county *i* in year *x*.

### 2.6. Health Data

The number of premature deaths from IHD attributable to PM_2.5_ exposure was estimated using baseline mortality rate data. The IHD baseline mortality rates were obtained from the Global Burden of Disease (GBD) dataset (http://ghdx.healthdata.org/, accessed on 1 June 2024) at a national scale. Based on the provincial-level study by Zhou et al. [42] and trends in GBD baseline mortality rates, it was assumed that annual variations in each province follow the national trend. Consequently, the provincial-level IHD mortality baseline for each year was calculated.

## 3. Methods

### 3.1. PM_2.5_ Concentration Retrieval

We constructed a machine learning model, XGBoost-PM25, for PM_2.5_ retrieval based on seamless 1 km resolution AOD data obtained from satellites, combined with meteorological, topographical, and socioeconomic features. XGBoost is an ensemble learning model that grows trees by continuously splitting features to fit the residuals from previous predictions, thereby reducing model bias. The structure score of XGBoost’s decision tree is given by Equation (2).

(2) $$obj_{b}\approx -\frac{1}{2}\sum_{j=1}^{J}\frac{G_{j}^{2}}{H_{j}+\alpha }+\omega J$$

where $obj_{b}$ represents the structure score of the new decision tree, *J* is the number of leaf nodes; $G_{j}$ and $H_{j}$ are known functions, ω is the complexity coefficient measuring the actual impact on model complexity with each additional leaf node, and α is the shrinkage function. The lower the structure score of the decision tree, the more reasonable its structure. We then used Shapley values to rank the contribution of input features and performed feature selection to construct the XGBoost-PM_2.5_ model dedicated to PM_2.5_ retrieval.

The Shapley value, derived from SHapley Additive exPlanations (SHAPs), is a method for explaining the predictions of machine learning models, introduced by Lundberg et al. [43] in 2017. For a given feature *j*, the Shapley value from all possible feature subsets *F* within the total feature set *S* is the difference between the outputs of two models: the first model includes the specific feature, and the second model does not. The Shapley value for the *j*-th feature of an observation *x* is measured by adding the *j*-th feature to all possible subsets, and the Shapley value is calculated according to the following formula (Equation (3)).

(3) $$SHAP_{j}\left(x\right)=\sum_{F\subseteq S-\{j\}}\frac{|F|!(|S|-|F|-1)!}{|S|!}\left[f_{x}\left(F\cup j\right)-f_{x}\left(F\right)\right]$$

In Equation (3), |F|! represents the number of permutations of features before the *j*-th feature, (|S|−|F|−1)! represents the number of permutations of features after the *j*-th feature, and |S|! is the total number of feature permutations. $f_{x}\left(F\right)$ denotes the predictive model *f* for sample *x*, with *F* being the subset that does not include the *j*-th feature, and $f_{x}\left(F\cup j\right)$ denotes the output of the same model including the *j*-th feature. In this way, the Shapley value assigns a score to each feature, reflecting its impact on the model’s prediction.

The average absolute Shapley values of each feature obtained using the above method are shown in Table 1. As indicated in Table 1, when the SHAP method ranks the features, the order is as follows: aerosol optical depth (AOD), fine mode fraction (FMF), temperature, digital elevation model (DEM), boundary layer height, population, slope (DEM-based), relative humidity, visibility, wind speed, precipitation, GDP, surface pressure, longitude, and latitude. By using the average absolute Shapley values to determine their contributions in the model evaluation, it can be observed that the cumulative average absolute Shapley values of the features, from the AOD down to precipitation, account for more than 95% of the total Shapley value (specifically, 96.99%). The features after precipitation were removed and normalized based on the magnitude of the mean absolute Shapley value, based on which weights were assigned to each input feature of the model.

Once the feature space is determined, the selection of hyperparameters significantly impacts the classification results. Hyperparameters were selected using the grid search method, which involves selecting a set of parameters from all the undetermined hyperparameters and then adjusting these parameters according to a specified step size, resulting in all possible hyperparameter combinations. By iterating through these combinations, the set of hyperparameters that yields the highest accuracy on the validation set is identified. The main hyperparameter settings for the final XGBoost-PM_2.5_ model are shown in Table 2.

The retrieval model was trained and validated using ground-level PM_2.5_ concentration data from 2012, 2017, and 2022. The accuracy of the model was evaluated using four indicators: coefficient of determination $R^{2}$, root mean square error (RMSE), mean absolute error (MAE), and relative percentage error (RPE). We assessed the retrieval accuracy of PM_2.5_ through 10-fold cross-validation (CV) and model fitting.

### 3.2. Estimation of Premature Mortality Due to IHD Attributable to PM_2.5_ Exposure

The premature mortality (ΔM) due to IHD attributable to PM_2.5_ exposure is calculated using Equation (4).

(4) $$\Delta M=y_{0}\times pop\times \frac{RR-1}{RR}$$

where $y_{0}$ represents the baseline mortality rate for IHD, which refers to the natural occurrence rate of deaths due to IHD without considering external factors (calculation method detailed in Section 2.6); pop represents the number of people in a specific age group exposed to ambient PM_2.5_ (with age ranges detailed in Table 3); and the RR represents the relative risk for a given PM_2.5_ concentration for the corresponding age group, calculated using the GEMM. GEMM, introduced by Burnett et al. [25] in 2018 and published in the Proceedings of the National Academy of Sciences (PNAS), evaluates premature mortality attributable to PM_2.5_ exposure based on a large body of epidemiological research. By constructing various concentration-response functions (CRFs), GEMM enhances the quality of outputs and the robustness of the model. The RR derived from GEMM is calculated using Equation (5).

(5) $$\left\{\begin{array}{c} RR=e^{A}\\ A=\frac{\theta log(1+\Delta C/\alpha )}{\left(1+B\right)}\\ B=e^{\left(-(C-\mu )/v\right)} \end{array}\right.$$

In Equation (5), ΔC represents the difference between ambient PM_2.5_ concentration and the baseline PM_2.5_ concentration. The GEMM model is constructed from the actual survey results on an annual scale, so the arithmetic mean of the four seasons in this study is the PM_2.5_ concentration for one year. The parameters α,θ,μ, and *v* are obtained through specific CRFs, with *A* and *B* representing calculated values. Since GEMM includes both global and region-specific sequences, this study uses age-specific model parameters based on Chinese cohort studies, as shown in Table 3. The baseline PM_2.5_ concentration is set at 2.4 μg/m^3^, referencing the exposure levels observed across all sequences in GEMM. According to Table 3, people of different age groups are affected differently by the same PM_2.5_ concentration. Premature mortality is influenced by the baseline mortality rate of IHD in the region as well as the PM_2.5_ concentration. The RR for IHD varies with age, with parameter θ decreasing as age increases. Given that θ is not a fixed value, a thousand Monte Carlo simulations were conducted to estimate the 95% confidence interval (CI) for premature mortality.

### 3.3. Assessment Method for Environmental Health Equity of PM_2.5_

The study adopts the Lorenz curve from economics to quantify the socioeconomic inequality in premature deaths caused by PM_2.5_ exposure. Traditionally, the Lorenz curve is used to describe the unequal distribution of wealth or income within a society [44,45]. In this study, we apply this concept to assess the inequitable distribution of premature deaths induced by PM_2.5_ exposure relative to the socioeconomic status of the exposed population. Due to the lack of direct income data, per capita GDP is used as a proxy indicator to reveal the relationship between socioeconomic status and health inequality.

**Figure 2:**
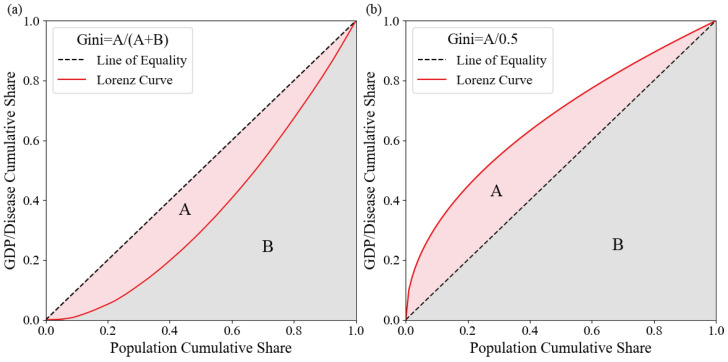
Gini coefficient illustration. Where A is the area between the Lorentz curve and the equality line, and B is the total area under the equality line. (**a**) When the Lorentz line is below the equality line. (**b**) When the Lorentz line is higher than the equality line.

For each grid cell within the region, we sort by per capita GDP and accumulate the corresponding population numbers and premature death counts to calculate cumulative percentages. The Lorenz curve is then plotted with the cumulative population percentage on the x-axis and the cumulative premature death percentage and GDP percentage on the y-axis. As shown in Figure 2, if the economic conditions of the exposed population are unrelated to PM_2.5_-induced premature deaths, the curve will be a 45-degree line (y = x). If the curve bulges to the right, it indicates that the exposed population in areas with low per capita GDP has a lower risk of PM_2.5_-related premature death, while those in high per capita GDP areas face higher health risks. Conversely, if the curve bulges to the left, it suggests that the exposed population in areas with low per capita GDP has a higher risk of PM_2.5_-induced premature death, while those in high-GDP areas have lower health risks. The further the curve deviates from the y = x line, the greater the inequality.

The degree of inequality is quantified using the Gini coefficient. As illustrated in Figure 2, the Gini coefficient is defined as the ratio of the area between the Lorenz curve and the line of perfect equality (45-degree line) to the total area under the line of perfect equality. The Gini coefficient ranges from 0 (complete equality) to 1 (complete inequality). This study calculates the Gini coefficient for each grid cell by sorting all grids by per capita GDP and computing the cumulative population percentage and cumulative premature death percentage using the following formula (Equation (6)).

(6) $$Gini=1-\sum_{i=1}^{n}\left(x_{i}-x_{i-1}\right)\left(y_{i}-y_{i-1}\right)$$

where n is the total number of grid cells in the study area, $x_{i}$ is the cumulative population percentage of the *i*-th grid cell, and $y_{i}$ is the cumulative percentage of PM_2.5_-induced premature deaths in the *i*-th grid cell. This study calculates the Gini coefficients for the premature deaths caused by PM_2.5_ pollution for IHD to explore the unequal distribution of health impacts among different socioeconomic groups.

## 4. Results and Discussion

### 4.1. Distribution Characteristics of PM_2.5_ Exposure in China

**Figure 3:**
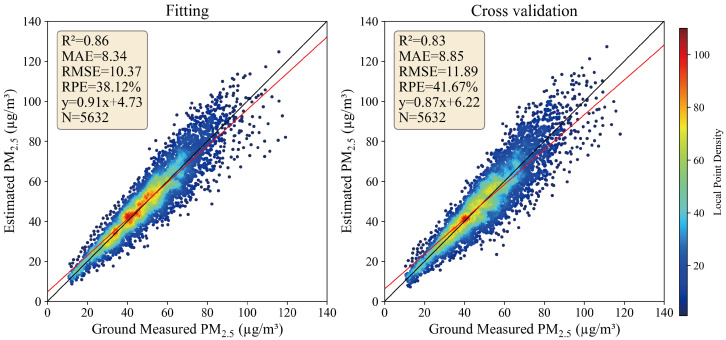
PM_2.5_ retrieval and ground measurement validation plot. The left figure shows the model fit validation plot, and the right figure shows the cross-validation plot.

The accuracy of the retrieved PM_2.5_ concentration data was evaluated using ground-measured PM_2.5_ concentrations, as shown in Figure 3. The model exhibited good consistency in both model fitting and 10-fold cross-validation (CV), with $R^{2}$ values of 0.86 and 0.83, respectively, and RMSE values of 10.37 μg/m^3^ and 11.89 μg/m^3^, indicating that the XGBoost-PM25 model has high accuracy in PM_2.5_ retrieval. The overall fitting accuracy was slightly higher than the 10-fold CV accuracy, indicating a slight overfitting. Compared to previous PM_2.5_ estimation studies, these metrics are within an acceptable range [46,47,48,49].

**Figure 4:**
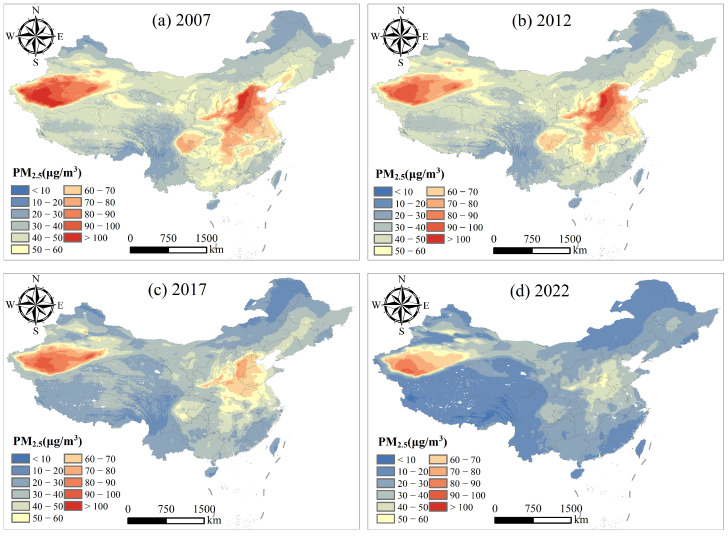
Distribution of PM_2.5_ exposure.

Using the XGBoost-PM25 model, we retrieved the spatial distribution of PM_2.5_ concentrations in China from 2007 to 2022, as shown in Figure 4. This figure displays the spatial distribution of PM_2.5_ concentrations in China at four anchor points: 2007, 2012, 2017, and 2022.

By comparing the maps for these years, significant changes in the spatial distribution of PM_2.5_ concentrations and their evolution over time can be observed. In 2007 (Figure 4a), PM_2.5_ concentrations were significantly higher in northern and central China, especially around Beijing and southern Xinjiang. The PM_2.5_ concentration in and around Beijing exceeded 100 μg/m^3^, indicating severe air pollution in that area. Other central regions, such as Henan and Shandong provinces, also had high PM_2.5_ concentrations, ranging from 50 to 100 μg/m^3^. In southeastern and southwestern China, PM_2.5_ concentrations were relatively low, with most areas ranging from 20 to 50 μg/m^3^. By 2012 (Figure 4b), areas with high PM_2.5_ concentrations had decreased, but PM_2.5_ concentrations in Beijing and its surrounding areas remained very high, with some areas exceeding 100 μg/m^3^. The high-concentration areas in central China had shrunk, and the coverage area had deecreaqsed. Southern Xinjiang still maintained high PM_2.5_ concentrations. However, PM_2.5_ concentrations in southeastern and southwestern regions remained low. The situation in 2017 (Figure 4c) showed further improvement. Although PM_2.5_ concentrations in Beijing and its surrounding areas were still high, they had decreased compared to previous years, mainly ranging from 70 to 100 μg/m^3^. High-concentration areas in central and eastern regions had also decreased. Xinjiang still maintained high PM_2.5_ concentrations, but the area had shrunk. Overall, air quality in eastern and southern coastal areas had improved, and PM_2.5_ concentrations continued to remain low. By 2022 (Figure 4d), PM_2.5_ concentrations in China had significantly decreased. PM_2.5_ concentrations in Beijing and its surrounding areas dropped to 50–70 μg/m^3^. High-concentration areas in central regions had significantly decreased, with most areas having PM_2.5_ concentrations between 20 and 50 μg/m^3^. PM_2.5_ concentrations in Xinjiang remained high but had improved compared to 2007. Most areas across the country saw significant reductions in PM_2.5_ concentrations, especially in eastern and southern coastal regions, where PM_2.5_ concentrations dropped to 10–20 μg/m^3^, indicating significant air quality improvement. PM_2.5_ concentration in China is high in winter and low in summer. From 2007 to 2022, the average value was 51.32 μg/m^3^ in winter and 25.41 μg/m^3^ in summer.

From 2007 to 2022, PM_2.5_ concentrations in China showed an overall downward trend (Table 4). Particularly in central and eastern regions, PM_2.5_ concentrations decreased by 33.23 μg/m^3^ and 29.70 μg/m^3^, respectively. This is related to a series of air protection plans China has implemented since 2013, including the Air Pollution Prevention and Control Action Plan (APPCAP), Three-Year Action Plan for Winning the Blue Sky Defense Battle (TAPWBSDB), and Action Plan for Continuous Improvement of Air Quality (APCIAQ). These measures have significantly improved air pollution conditions in recent years. PM_2.5_ concentrations in eastern and southern coastal regions have remained low due to industrial restructuring and environmental management measures. Although PM_2.5_ concentrations in Xinjiang remain high, the overall trend also shows improvement. These changes reflect significant achievements in China’s air pollution control efforts, with the national average PM_2.5_ concentration in 2022 dropping by as much as 46.93% compared to 2007.

### 4.2. Distribution of Premature Deaths Due to IHD Attributed to PM_2.5_ Exposure

**Figure 5:**
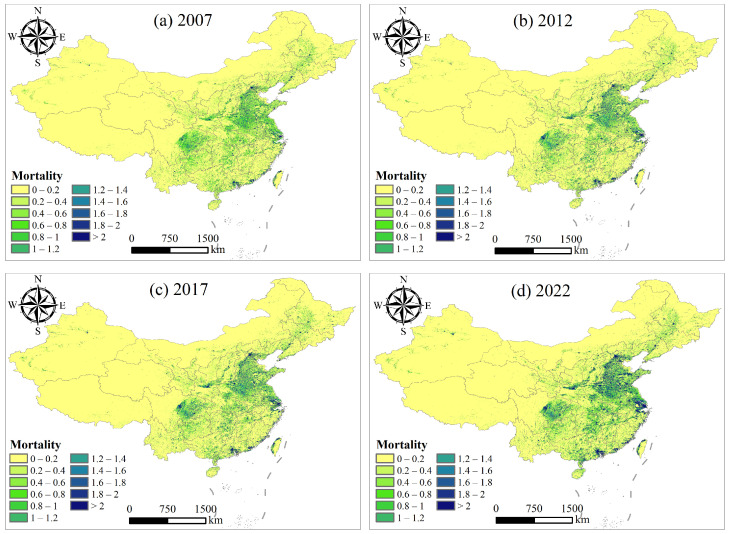
Distribution of premature deaths due to IHD attributable to PM_2.5_ exposure.

The number of premature deaths due to IHD attributed to PM_2.5_ exposure was obtained using the GEMM model, which incorporates PM_2.5_ concentrations, population density, resident age distribution, and baseline mortality rate data. The distribution in China for the years 2007, 2012, 2017, and 2022 is shown in Figure 5. In the figure, premature mortality represents the number of deaths per 1 km^2^ (shown as one grid cell in the figure). Given the sparse population density in some areas, the number of premature deaths can be a decimal. The direct rounding of these numbers would reduce the accuracy of the study; therefore, even if the results are decimal, they are retained. Such premature death counts can be interpreted as indicating that, while there may be no deaths within a 1 km^2^ area, there could be cases of premature deaths within a larger spatial range (e.g., 10 km^2^).

In 2007 (Figure 5a), the premature mortality from IHD attributed to PM_2.5_ exposure was higher in central and eastern regions. These areas, including provinces such as Henan, Shandong, Jiangsu, and Anhui, had premature mortality ranging from 0.4 to 1.2, with some local areas exceeding 1.2. In contrast, western regions such as Xinjiang in the northwest and Tibet in the southwest had lower premature mortality, mostly between 0 and 0.4. The distribution of premature mortality mirrored the spatial distribution of PM_2.5_ concentrations in 2007, while also accounting for population density. Regions with high pollution levels and high population densities exhibited relatively high premature mortality. By 2012 (Figure 5b), the premature mortality in eastern and central regions had increased, particularly around Beijing and the Yangtze River Delta, with some areas experiencing an increase in premature mortality to between 1.2 and 1.4. In contrast, the western regions saw little change and remained at lower levels. In 2017 (Figure 5c), the premature mortality continued to rise in the eastern and central regions, with some areas exceeding 1.4. Meanwhile, eastern and southern coastal regions, such as Guangdong and Fujian, despite having relatively lower PM_2.5_ concentrations, experienced a significant increase in premature mortality due to high population densities. By 2022 (Figure 5d), despite a significant reduction in PM_2.5_ concentrations across most regions of the country (as shown in Figure 4), the premature mortality in eastern and central regions remained high. According to the GEMM model, people of different ages have different premature death rates when facing the same PM_2.5_ concentration (see Equation (5) and Table 3). The GEMM model indicates that this result is due to population aging and an increase in baseline mortality rates. This highlights the need to consider other health impact factors alongside improving air quality.

Statistics on the number of premature deaths from and rates of IHD attributed to PM_2.5_ pollution in various regions are shown in Table 5, with 95% confidence intervals from 1000 Monte Carlo simulations provided in brackets. Table 5 indicates that, despite some regional fluctuations, the overall number of premature deaths has shown an upward trend from 2007 to 2022, even as PM_2.5_ concentrations have generally decreased during this period. This increase is driven by changes in the age structure of the population and baseline mortality rates. Health risks in the eastern and central regions are particularly pronounced, with 262,026 and 140,963 premature deaths in 2022, respectively. Nationwide, the number of premature deaths from IHD due to PM_2.5_ exposure increased from 819,287 in 2007 to 870,138 in 2022. Furthermore, during the period from 2007 to 2022, the premature mortality rate in China showed a very slight change, decreasing from 61.96/$10^{5}$ to 61.66/$10^{5}$ (a 0.48% decrease), while the PM_2.5_ concentration dropped significantly by 46.93%. During this period, the premature mortality rate in the northeastern and northern regions increased by 5.57/$10^{5}$ and 1.04/$10^{5}$, respectively. The significant reduction in PM_2.5_ levels alongside stable or even increasing premature mortality rates suggests that population aging is becoming a more prominent factor in IHD-related premature deaths attributed to PM_2.5_ exposure. This suggests that while further improvements in air quality are essential, there is also a need to strengthen health monitoring and protection measures for high-risk populations.

### 4.3. Analysis of Environmental Equity in Premature Mortality Due to IHD Attributed to PM_2.5_ Exposure

**Figure 6:**
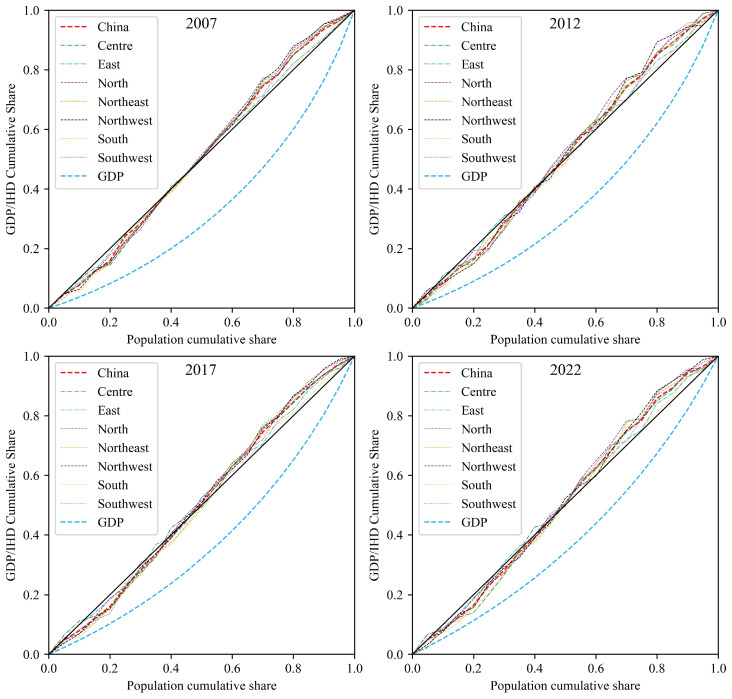
Lorenz curves of premature mortality from IHD and GDP due to PM_2.5_ exposure.

Lorenz curves were plotted to analyze the differences in premature mortality from IHD among different economic groups in China (Figure 6). Figure 6 shows the Lorenz curves for the cumulative share of premature mortality from IHD across different economic groups and regions in China at four anchor points: 2007, 2012, 2017, and 2022. The horizontal axis represents the population’s cumulative share, while the vertical axis represents the cumulative share of premature mortality from IHD/GDP. The solid black line represents the line of absolute equality.

The Lorenz curves for different regions in China at the four time points clearly deviate from the diagonal, indicating an unequal distribution of premature mortality from IHD. From 2007 to 2022, the Lorenz curves for GDP shifted closer to the line of absolute equality, suggesting that economic inequality decreased, although some inequality remains. The cumulative share of premature mortality from IHD did not change significantly during this period, remaining relatively balanced. Among the regions, the distribution of premature mortality from IHD in the eastern and southern regions is closer to the line of absolute equality, especially in the southern region, possibly benefiting from better economic development and medical resources. In contrast, the northern and northeastern regions exhibit varying degrees of inequality in premature mortality from IHD, reflecting disparities in economic and healthcare security within these regions. The northwestern region deviates the most from the line of absolute equality, indicating the poorest environmental justice among all regions in China.

Although the GDP gap has been gradually narrowing over time, the cumulative share of premature mortality from IHD has been consistently concentrated among the middle-income group (represented by the steepest slope of the Lorenz curve when the population cumulative share is between 0.4 and 0.6 in Figure 6). This phenomenon might stem from unique health risks faced by the middle-income group, such as high work pressure and unhealthy lifestyles. The high-income and low-income groups have relatively lower cumulative shares of premature mortality from IHD. High-income groups likely benefit from better medical conditions and health awareness, while low-income groups might have lower IHD mortality due to lower economic activity levels and higher physical labor intensity.

The Gini coefficients for different regions at the same time points are listed in Table 6. These coefficients allow for further analysis of environmental equity in premature mortality from IHD across different regions in China. A Gini coefficient closer to 0 indicates a more even distribution, while a value closer to 1 indicates a higher degree of inequality. From 2007 to 2022, the national Gini coefficient for IHD remained relatively low and stable, between 0.025 and 0.029. However, there are regional disparities in environmental equity regarding premature mortality from IHD. The southern and northeastern regions exhibited higher environmental equity during this period, whereas the central and northwestern regions were relatively unequal. The order of environmental equity from high to low is as follows: South: 0.019; East: 0.023; Northeast: 0.026; North: 0.028; Southwest: 0.029; Center: 0.030; Northwest: 0.035. Therefore, public health policies should particularly focus on the central and northwestern regions, implementing measures to improve the fairness in the distribution of premature mortality from IHD.

### 4.4. Strengths and Limitations of This Study

Although several studies have explored the impact of long-term PM_2.5_ exposure on IHD, this study offers significant advantages in both precision and scope. Many studies are limited to specific regions and short-term changes, such as De et al.’s [20] research on Rome, Italy, over two years and Yu et al. [16] on Guangzhou, China, over three years. Maji et al. [9] estimated premature deaths due to PM_2.5_ exposure in China from 2015 to 2019, with a spatial resolution of only 0.1° and a temporal span of only five years. Their conclusions primarily highlighted the need to reduce premature mortality to combat air pollution. In contrast, this study achieved notable improvements in spatial resolution, coverage, and temporal span. It not only confirms the progress China has made in air pollution control over the past fifteen years but also reveals the increasing impact of the population’s aging on IHD-related premature deaths attributable to PM_2.5_ exposure. Furthermore, by comparing premature mortality across different economic groups, this study identifies that the risk of premature deaths due to PM_2.5_ exposure is more pronounced among middle-income populations.

However, this study has some limitations that require further refinement. The research primarily used the Gini coefficient to analyze health inequalities among different income groups but did not thoroughly investigate the moderating effects of other socioeconomic factors, such as educational attainment and occupational exposure, on the relationship between PM_2.5_ exposure and health outcomes. Additionally, using GDP as a proxy for income level may introduce some inaccuracies, suggesting that future research should employ more precise data. Beyond PM_2.5_, future studies should also consider the combined health effects of various air pollutants (e.g., NOx, SO2) and explore the interactions between different pollutants to comprehensively assess the health risks posed by air pollution.

## 5. Conclusions

This study utilized the XGBoost-PM_2.5_ machine learning model and the GEMM model to obtain PM_2.5_ exposure levels and the associated premature mortality from IHD in China from 2007 to 2022 and to explore the resulting environmental equity issues. The results show significant regional differences in PM_2.5_ concentrations across China, with higher levels in the central and eastern regions (53.55 μg/m^3^ and 44.78 μg/m^3^, respectively) compared to lower levels in the northeastern and southwestern regions (33.81 μg/m^3^ and 29.68 μg/m^3^, respectively). Overall, PM_2.5_ concentrations in China decreased from 47.41 μg/m^3^ in 2007 to 25.16 μg/m^3^ in 2022, with the most notable reduction in the central region (33.23 μg/m^3^). However, the number of premature IHD deaths attributable to PM_2.5_ exposure increased from 819 thousand in 2007 to 870 thousand in 2022. This increase is associated with population growth and aging during this period, despite the decline in PM_2.5_ concentrations. The Lorenz curves reveal that the share of IHD premature mortality among the middle-income group is higher than that of the high-income or low-income groups. This phenomenon may be due to unique health risks faced by the middle-income group. The Gini coefficients indicate that environmental equity across regions in China ranks from highest to lowest as follows: South, East, Northeast, North, Southwest, Center, and Northwest. The methods and findings of this study provide a scientific basis for developing more effective and targeted air pollution control policies, highlighting the need to consider environmental health equity alongside improvements in air quality.

## Figures and Tables

**Table 1:** Average absolute Shapley values of feature factors in the initial XGBoost model.

Feature	Shapley	Feature	Shapley
AOD	8.63	Visibility	0.54
FMF	4.11	Wind speed	0.50
Temperature	2.05	Precipitation	0.48
DEM	1.56	GDP	0.31
Boundary layer height	1.21	Surface pressure	0.18
Population	1.12	Longitude	0.10
Slope	0.83	Latitude	0.08
Relative humidity	0.59		

**Table 2:** Hyperparameter settings for the SHAP-XGBoost model for PM_2.5_ retrieval.

Hyperparameter	Value	Hyperparameter	Value
num_boosting_rounds	180	gamma	0.02
learning_rate	0.05	min_child_weight	2
max_depth	10	subsample	1
max_delta_step	0	scale_pos_weight	1

**Table 3:** GEMM-based RR Calculation Parameters for IHD.

Name	Age Range	θ	Standard Deviation of θ	α	μ	ν
IHD	≥25	0.2969	0.01787	1.9	12	40.2
	25–29	0.5070	0.02458	1.9	12	40.2
	30–34	0.4762	0.02309	1.9	12	40.2
	35–39	0.4455	0.02160	1.9	12	40.2
	40–44	0.4148	0.02011	1.9	12	40.2
	45–49	0.3841	0.01862	1.9	12	40.2
	50–54	0.3533	0.01713	1.9	12	40.2
	55–59	0.3226	0.01564	1.9	12	40.2
	60–64	0.2919	0.01415	1.9	12	40.2
	65–69	0.2612	0.01266	1.9	12	40.2
	70–74	0.2304	0.01117	1.9	12	40.2
	75–79	0.1997	0.00968	1.9	12	40.2
	≥80	0.1536	0.00740	1.9	12	40.2

**Table 4:** Changes in PM_2.5_ concentrations (μg/m^3^) in China from 2007 to 2022.

Region	2007	2012	2017	2022
China	47.41	45.91	35.95	25.16
East	56.84	53.13	42.01	27.14
Northeast	39.16	40.44	32.56	23.09
Centre	67.09	66.17	47.08	33.86
North	43.67	42.82	33.05	23.69
South	44.97	38.65	31.59	21.38
Northwest	52.99	50.89	41.42	31.58
Southwest	38.03	37.24	27.66	15.80

**Table 5:** Number of premature deaths and rate (/$10^{5}$) due to IHD attributable to PM_2.5_ exposure in China (2007–2022).

Region	2007	2012	2017	2012
China	819,287, 61.96 (720,973, 917,601)	833,170, 61.53 (733,190, 933,150)	858,290, 61.75 (755,295, 961,285)	870,138, 61.66 (765,721, 974,555)
East	242,901, 57.28 (213,753, 272,049)	251,996, 56.60 (221,757, 282,236)	259,496, 57.01 (228,356, 290,635)	262,026, 55.15 (230,583, 293,469)
Northeast	67,589, 60.90 (59,478, 75,699)	66,902, 61.37 (58,874, 74,930)	68,849, 64.29 (60,587, 77,111)	69,885, 66.47 (61,499, 78,272)
Centre	138,096, 57.47 (121,524, 154,667)	132,607, 56.04 (116,694, 148,520)	137,384, 55.17 (120,898, 153,870)	140,963, 54.86 (124,047, 157,878)
North	94,431, 79.04 (83,100, 105,763)	101,042, 81.82 (88,917, 113,167)	104,176, 81.47 (91,675, 116,677)	106,734, 80.08 (93,926, 119,542)
South	91,838, 54.31 (80,818, 102,859)	101,191, 55.54 (89,048, 113,334)	103,699, 53.67 (91,255, 116,142)	103,260, 51.58 (90,869, 115,651)
Northwest	58,169, 62.15 (51,188, 65,149)	59,607, 61.73 (52,454, 66,760)	61,336, 61.27 (53,976, 68,696)	62,653, 61.22 (55,135, 70,172)
Southwest	126,264, 58.04 (111,112, 141,415)	119,826, 56.72 (105,447, 134,205)	123,352, 56.21 (108,549, 138,154)	124,618, 55.81 (109,664, 139,572)

**Table 6:** Gini coefficients for premature mortality from IHD; cumulative shares in China, 2007–2022.

Region	2007	2012	2017	2022
China	0.026	0.029	0.028	0.025
East	0.028	0.023	0.019	0.023
Northeast	0.026	0.032	0.024	0.021
Centre	0.024	0.034	0.036	0.027
North	0.033	0.026	0.031	0.023
South	0.018	0.023	0.016	0.017
Northwest	0.034	0.038	0.040	0.028
Southwest	0.023	0.027	0.032	0.034

## Data Availability

All publicly accessible online data have been listed in the text, and the code for this study is available upon request from the first author.
